# Supplementary material for: SynAPSeg: A novel dataset and image analysis framework for deep learning-based synapse detection and quantification
Source: PLoS Comput Biol. 2026 Jul 29;22(7):e1014571. doi: 10.1371/journal.pcbi.1014571 (PMC13432752; doi:10.1371/journal.pcbi.1014571)
Supplement: S1 Table — Table with the details related to sample preparation of imaging data used for training and validation of models. (DOCX) [file pcbi.1014571.s009.docx]

|  | **Dataset alias** | **nD** | **Marker** | **Labeling modality** | **Annotation type** | **Sample preparation** | **Denoising** | **Z**  **(um)** | **YX**  **(um)** |
| --- | --- | --- | --- | --- | --- | --- | --- | --- | --- |
| 0 | 2D_Tissue_vGatCre_PSD95GFP | 2D | PSD95-GFP | transgenic (vgat-cre;PSD95-GFP) | ROI | tissue | n2v | 0.3 | 0.071 |
| 1 | 2D_Tissue_vGatCre_PSD95GFP | 2D | PSD95-GFP | transgenic (vgat-cre;PSD95-GFP) | ROI | tissue | n2v | 0.3 | 0.071 |
| 2 | 2D_Tissue_vGatCre_PSD95GFP | 2D | PSD95-GFP | transgenic (vgat-cre;PSD95-GFP) | ROI | tissue | none | 0.3 | 0.071 |
| 3 | 2D_Tissue_vGatCre_PSD95GFP | 2D | PSD95-GFP | transgenic (vgat-cre;PSD95-GFP) | ROI | tissue | none | 0.3 | 0.071 |
| 4 | 2D_Tissue_vGatCre_PSD95GFP | 2D | PSD95-GFP | transgenic (vgat-cre;PSD95-GFP) | ROI | tissue | none | 0.3 | 0.071 |
| 5 | 2D_Culture_Transfection_PSD95 | 2D | PSD95-FingR-GFP | transfection | full | in vitro | none | 0.5 | 0.071 |
| 6 | 2D_Culture_Transfection_PSD95 | 2D | PSD95-FingR-GFP | transfection | full | in vitro | none | 0.5 | 0.071 |
| 7 | 2D_Culture_Transfection_PSD95 | 2D | PSD95-FingR-GFP | transfection | full | in vitro | none | 0.5 | 0.071 |
| 8 | 2D_Culture_Transfection_PSD95 | 2D | PSD95-FingR-GFP | transfection | full | in vitro | none | 0.5 | 0.071 |
| 9 | 2D_Culture_Transfection_PSD95 | 2D | PSD95-FingR-GFP | transfection | full | in vitro | none | 0.5 | 0.071 |
| 10 | 2D_Culture_Transfection_PSD95 | 2D | PSD95-FingR-GFP | transfection | full | in vitro | none | 0.5 | 0.071 |
| 11 | 2D_Culture_Transfection_PSD95 | 2D | PSD95-FingR-GFP | transfection | full | in vitro | n2v | 0.5 | 0.071 |
| 12 | 2D_Culture_Transfection_PSD95 | 2D | PSD95-FingR-GFP | transfection | full | in vitro | none | 0.5 | 0.071 |
| 13 | 2D_Culture_Transfection_PSD95 | 2D | PSD95-FingR-GFP | transfection | full | in vitro | none | 0.5 | 0.071 |
| 14 | 2D_Culture_Transfection_PSD95 | 2D | PSD95-FingR-GFP | transfection | full | in vitro | none | 0.5 | 0.071 |
| 15 | 2D_Culture_Transfection_PSD95 | 2D | PSD95-FingR-GFP | transfection | full | in vitro | none | 0.5 | 0.071 |
| 16 | 2D_Culture_Transfection_PSD95 | 2D | PSD95-FingR-GFP | transfection | full | in vitro | none | 0.5 | 0.071 |
| 17 | 2D_Culture_IHC | 2D | Traf3 (1:400, Proteintech #66310-1-Ig) | IHC | ROI | in vitro | none | 0.5 | 0.071 |
| 18 | 2D_Culture_IHC | 2D | Traf3 (1:400, Proteintech #66310-1-Ig) | IHC | ROI | in vitro | none | 0.5 | 0.071 |
| 19 | 2D_Culture_IHC | 2D | Homer | IHC | ROI | in vitro | none | 0.5 | 0.071 |
| 20 | 2D_Culture_IHC | 2D | Traf3 (1:400, Proteintech #66310-1-Ig) | IHC | ROI | in vitro | none | 0.5 | 0.071 |
| 21 | 2D_Culture_IHC | 2D | Vglut1 (1:500, SYSY #135304) | IHC | ROI | in vitro | none | 0.5 | 0.071 |
| 22 | 2D_Culture_IHC | 2D | Homer (SYSY #160002) | IHC | full | in vitro | none | 0.5 | 0.071 |
| 23 | 2D_Culture_Transfection_PSD95 | 2D | PSD95-FingR-GFP | transfection | ROI | in vitro | none | 0.5 | 0.085 |
| 24 | 2D_Culture_Transfection_PSD95 | 2D | PSD95-FingR-GFP | transfection | full | in vitro | none | 0.5 | 0.085 |
| 25 | 2D_Culture_Transfection_PSD95 | 2D | Traf3-GFP | transfection | Full | in vitro | none | 0.5 | 0.071 |
| 26 | 2D_Culture_Transfection_PSD95 | 2D | PSD95-mCh | transfection | full | in vitro | none | 0.5 | 0.071 |
| 0 | 3D_Culture_transfection_PSD95 | 3D | PSD95-fingR | transfection | full | in vitro | none | 0.19 | 0.071 |
| 1 | 3D_Culture_IHC | 3D | Traf3-GFP | IHC | ROI | in vitro | none | 0.33 | 0.071 |
| 2 | 3D_Culture_IHC | 3D | Homer | IHC | ROI | in vitro | none | 0.22 | 0.071 |
| 3 | 3D_Culture_IHC | 3D | Gephyrin (1:1000, SYSY #147011) | IHC | full | in vitro | none | 0.4 | 0.071 |
| 4 | 3D_Culture_IHC | 3D | PSD95 | IHC | full | in vitro | none | 0.4 | 0.071 |
| 5 | 3D_Tissue_vGatCre_PSD95GFP | 3D | PSD95-GFP | transgenic (vgat-cre;PSD95-GFP) | full | in vitro | none | 0.23 | 0.085 |
| 6 | 3D_Tissue_Geph | 3D | DIO-GephFingR-mScarlet | AAV, vgat-cre mouse | full | tissue | none | 0.28 | 0.1 |
| 7 | 3D_Tissue_vGatCre_PSD95GFP | 3D | PSD95-GFP | transgenic (vgat-cre;PSD95-GFP) | full | tissue | n2v | 0.46 | 0.071 |
| 8 | 3D_Tissue_vGatCre_PSD95GFP | 3D | PSD95-GFP | transgenic (vgat-cre;PSD95-GFP) | full | tissue | none | 0.46 | 0.071 |
| 9 | 3D_Tissue_vGatCre_PSD95GFP | 3D | PSD95-GFP | transgenic (vgat-cre;PSD95-GFP) | full | tissue | none | 0.46 | 0.071 |
| 10 | 3D_Tissue_vGatCre_PSD95GFP | 3D | PSD95-GFP | transgenic (vgat-cre;PSD95-GFP) | full | tissue | n2v | 0.46 | 0.071 |
| 11 | 3D_Tissue_vGatCre_PSD95GFP | 3D | PSD95-GFP | transgenic (vgat-cre;PSD95-GFP) | full | tissue | none | 0.46 | 0.071 |
| 12 | 3D_Tissue_EN_PSD95 | 3D | PSD95-GFP | AAV-cre, PSD95-GFP mouse | ROI | tissue | none | 0.46 | 0.085 |
| 13 | 3D_Tissue_EN_PSD95 | 3D | PSD95-GFP | AAV-cre, PSD95-GFP mouse | ROI | tissue | none | 0.46 | 0.085 |
| 0 | Benchmark1 | 2D | PSD95-FingR-GFP | transfection | full | in vitro | none | 0.5 | 0.071 |
| 1 | Benchmark2 | 2D | Vglut1 (1:500, SYSY #135304) | IHC | full | in vitro | none | 0.5 | 0.071 |
| 2 | Benchmark3 | 3D | PSD95-GFP | transgenic (vgat-cre;PSD95-GFP) | full | tissue | none | 0.46 | 0.071 |
