# Supplementary material for: SynAPSeg: A novel dataset and image analysis framework for deep learning-based synapse detection and quantification
Source: PLoS Comput Biol. 2026 Jul 29;22(7):e1014571. doi: 10.1371/journal.pcbi.1014571 (PMC13432752; doi:10.1371/journal.pcbi.1014571)
Supplement: S2 Table — Table with statistical details for all tests performed and information about the N for each experiment. (DOCX) [file pcbi.1014571.s010.docx]

| **Main Figures** | **Test** | **Test statistics** | **P-value** | **Sample information** |
| --- | --- | --- | --- | --- |
| 1C | T-test (Precision) | StarDist_custom vs cellpose_custom  T = 3.5992 | 0.0009 | DF = 78 |
|  |  | StarDist_custom vs cellpose_default  T = 15.0012 | < 4E-24 |  |
|  |  | StarDist_custom vs deepd3  T = 21.4798 | < 3E-33 |  |
|  |  | StarDist_custom vs stardist_default  T = 19.4129 | < 2E-30 |  |
|  |  | StarDist_custom vs threshold + watershed  T = 14.2980 | < 5E-23 |  |
|  | T-test (Recall) | StarDist_custom vs cellpose_custom  T = 1.9851 | 0.0542 |  |
|  |  | StarDist_custom vs cellpose_default  T = 24.9175 | < 2E-37 |  |
|  |  | StarDist_custom vs deepd3  T = 17.7074 | < 2E-28 |  |
|  |  | StarDist_custom vs stardist_default  T = 17.8332 | < 2E-28 |  |
|  |  | StarDist_custom vs threshold + watershed  11.1852 | < 2E-17 |  |
|  | T-test (f1) | StarDist_custom vs cellpose_custom  T = 3.4433 | 0.0012 |  |
|  |  | StarDist_custom vs cellpose_default  T =23.0942 | <2E-35 |  |
|  |  | StarDist_custom vs deepd3  T = 22.3959 | <8E-35 |  |
|  |  | StarDist_custom vs stardist_default  T =20.7158 | <1E-32 |  |
|  |  | StarDist_custom vs threshold + watershed  T =14.0788 | <9E-23 |  |
| 1E | T-test (Precision) | StarDist_custom vs cellpose_custom  T = 3.8741 | 0.0183 | DF=7 |
|  |  | StarDist_custom vs cellpose_default  T =6.5654 | 0.0024 |  |
|  |  | StarDist_custom vs deepd3  T =6.8175 | 0.0024 |  |
|  |  | StarDist_custom vs stardist_default  T =4.0609 | 0.0180 |  |
|  |  | StarDist_custom vs threshold + watershed  T =6.0272 | 0.0026 |  |
|  | T-test (Recall) | StarDist_custom vs cellpose_custom  T =2.7813 | 0.0372 |  |
|  |  | StarDist_custom vs cellpose_default  T =7.4768 | 0.0011 |  |
|  |  | StarDist_custom vs deepd3  T =5.6758 | 0.0028 |  |
|  |  | StarDist_custom vs stardist_default  T =10.1547 | 0.0003 |  |
|  |  | StarDist_custom vs threshold + watershed  T =6.2228 | 0.0022 |  |
|  | T-test (f1) | StarDist_custom vs cellpose_custom  T =3.3656 | 0.0200 |  |
|  |  | StarDist_custom vs cellpose_default  T =6.6734 | 0.0016 |  |
|  |  | StarDist_custom vs deepd3  T =6.5630 | 0.0016 |  |
|  |  | StarDist_custom vs stardist_default  T =8.3043 | 0.0011 |  |
|  |  | StarDist_custom vs threshold + watershed  T =6.0543 | 0.0019 |  |
| 2B | One-sample t-test (Mean IoU score) | Dataset 1  T =-2.2657 | 0.0861 | DF=4 |
|  |  | Dataset 2  T =-2.3595 | 0.0777 |  |
|  |  | Dataset 3  T =2.26485 | 0.0862 |  |
| 2I | One-sample t-test (Dataset 1: precision, recall, f1) | Precision  T =6.2372 | 0.0034 |  |
|  |  | Recall  T =1.2910 | 0.2663 |  |
|  |  | F1  T =6.6948 | 0.0026 |  |
| 2J | One-sample t-test (Dataset 2: precision, recall, f1) | Precision  T =1.2115 | 0.2910 |  |
|  |  | Recall  T =-1.2910 | 0.2663 |  |
|  |  | F1  T =-0.4517 | 0.6749 |  |
| 2K | One-sample t-test (Dataset 3: precision, recall, f1) | Precision  T =-1.1952 | 0.2980 |  |
|  |  | Recall  T =-0.4564 | 0.6718 |  |
|  |  | F1  T =-1.5403 | 0.1983 |  |
| 4D | **LMM effect of sex comparisons** | Dependent Variables: Density, Size, Intensity |  |  |
|  | Within 3-Month Subset: Sex | Density  Coefficient = -14290 | 0.280 | N = 2 female 4 male 3-month |
|  |  | Size  Coefficient =2.586 | 0.186 |  |
|  |  | Intensity  Coefficient =-547.7 | 0.520 |  |
|  | Within Female Subset: Age | Density  Coefficient = 11171 | 0.505 | N = 2 3-month and 4 12-month females |
|  |  | Size  Coefficient =-0.235 | 0.845 |  |
|  |  | Intensity  Coefficient = -111.8 | 0.905 |  |
|  | Overall Cohort Model: Sex | Density  Coefficient = 79.52 | 0.995 | N = 6 3-month (2 female 4 male) and 5 12-month (4 female 1 male) |
|  |  | Size  Coefficient =1.421 | 0.299 |  |
|  |  | Intensity  Coefficient =-1963 | 0.039 |  |
|  | Overall Cohort Model: Age | Density  Coefficient = -3200 | 0.813 |  |
|  |  | Size  Coefficient = 0.930 | 0.497 |  |
|  |  | Intensity  Coefficient = 1304 | 0.171 |  |
| 4E | **Two-Way ANOVA (Puncta density)** |  |  | N = 6 3-month (2 female 4 male) and 5 12-month (4 female 1 male) |
|  | age * region | F(11.0, 108) = 0.3175 | 0.9807 |  |
|  | region | F(11.0, 108) = 25.3367 | < 0.0001 |  |
|  | age | F(11.0, 108) = 0.4456 | 0.5058 |  |
|  | Šídák's multiple comparisons, paired T-test | df=5 |  |  |
|  | **Two-Way ANOVA (Puncta size)** |  |  |  |
|  | age * region | F(11.0, 108) = 0.1532 | 0.9992 |  |
|  | region | F(11.0, 108) = 14.5583 | < 0.0001 |  |
|  | age | F(11.0, 108) = 14.3116 | 0.0003 |  |
|  | Šídák's multiple comparisons, paired T-test | df=5 |  |  |
|  | **Two-Way ANOVA (Puncta intensity)** |  |  |  |
|  | age * region | F(11.0, 108) = 0.1608 | 0.9990 |  |
|  | region | F(11.0, 108) = 1.7421 | 0.0736 |  |
|  | age | F(11.0, 108) = 1.7438 | 0.1894 |  |
|  | Šídák's multiple comparisons, Welch's T-test | df=5 |  |  |
| 5 | **Linear regression models (within 3-month sex comparison)** |  |  | N = 2 female 4 male 3-month |
|  | Density | Coefficient = -0.0388 | 0.849 |  |
|  | Size | Coefficient = 0.0016 | 0.928 |  |
|  | PSD95 intensity | Coefficient = -892.1 | 0.259 |  |
|  | PV ROI intensity | Coefficient = -1335 | 0.238 |  |
|  | **Linear regression models (within females age comparison)** |  |  | N = 2 3-month and 4 12-month females |
|  | Density | Coefficient = 0.4727 | 0.017 |  |
|  | Size | Coefficient = 0.0312 | 0.26 |  |
|  | PSD95 intensity | Coefficient = 1489 | 0.26 |  |
|  | PV ROI intensity | Coefficient = 2769 | 0.01 |  |
| 5C | Welch’s T-test (PV intensity) | t=-3.2447, df=14.9854 | 0.0054 | N = 11 3-month (N=5 females, 6 males) and 6 12-month: (5 females, 1 male) |
| 5E | Welch’s T-test (PSD95 size) | t=-0.6892, df=6.2599 | 0.5154 | N = 11 3-month (N=5 females, 6 males) and 6 12-month: (5 females, 1 male) |
| 5F | Mann Whiteney test (PSD95 intensity) | U=18 | 0.1490 | N = 11 3-month (N=5 females, 6 males) and 6 12-month: (5 females, 1 male) |
| 5G | Welch’s T-test (PSD95 linear density) | t=-3.2562, df=9.8126 | 0.0088 | N = 11 3-month (N=5 females, 6 males) and 6 12-month: (5 females, 1 male) |
|  | **Linear regression model (density ~ age * PSD95 ROI intensity)** |  |  |  |
|  | Age | Coefficient = 2.8495 | 0.008 |  |
|  | PSD95 ROI intensity | Coefficient = 0.0002 | 0.026 |  |
|  | Age x PSD95 ROI intensity | Coefficient = -0.0004 | 0.019 |  |
| **Supplementary Figures** | | | | |
| S3A | One-sample t-test (Count) | Dataset 1  T =-.3641 | 0.0220 | DF=4 |
|  |  | Dataset 2  T =0.7109 | 0.5164 |  |
|  |  | Dataset 3  T =2.0566 | 0.1089 |  |
| S3B | One-sample t-test (Size) | Dataset 1  T =-2.3591 | 0.0777 |  |
|  |  | Dataset 2  T =0.7097 | 0.5171 |  |
|  |  | Dataset 3  T =0.7989 | 0.4691 |  |
| S3C | One-sample t-test (Intensity) | Dataset 1  T = -0.8793 | 0.4289 |  |
|  |  | Dataset 2  T =-1.819 | 0.1430 |  |
|  |  | Dataset 3  T =3.9982 | 0.0161 |  |
| S6A | Mann Whitney test (Dendrite volume) | U=26 | 0.5249 | N = 11 3-month (N=5 females, 6 males) and 6 12-month: (5 females, 1 male) |
| S6A | Mann Whitney test (Dendrite length) | U=38 | 0.6605 | N = 11 3-month (N=5 females, 6 males) and 6 12-month: (5 females, 1 male) |
| S6B | Pearson correlation (PV intensity x PSD95 linear density) | 3-month r=0.3192 | 0.0039 | N = 80 3-month, and 33 12-month dendrites (dendrites per animal: 5-11, average: 7) |
|  |  | 12-month r=0.5084 | 0.0025 |  |
|  | Fisher r-to-z | z=1.0675 | 0.2857 |  |
| S6E | Pearson correlation (Nearest neighbor analysis of puncta intensity) | 3-month: r=0.7039 | < 1E-300 | N = 5515 3-month and 1856 12-month PSD95 puncta |
|  |  | 12-month: r=0.7119 | < 1E-286 |  |
|  | Fisher r-to-z | z=-0.0700 | 0.9400 |  |
| S6F | Pearson correlation (Randomly sampled neighbors) | 3-month: r=0.0094 | 0.4859 | N = 5515 3-month and 1856 12-month PSD95 puncta |
|  |  | 12-month: r=-0.0275 | 0.2359 |  |
| S7C | Pearson correlation (Synapse density vs Distance from soma) | 3-month: r=0.0033 | 0.9230 | N = 859 3-month and 492 12-month dendritic segments |
|  |  | 12-month: r=0.2497 | < 2E-8 |  |
|  | Fisher r-to-z | z=4.44 | < 9E-6 |  |
| S7E | Mann Whitney test (PSD95 density in proximal-distal bins) | proximal (< 40 um)  U=8 | 0.0103 | N = 11 3-month and 6 12-month |
|  |  | Intermediate  U=7 | 0.0071 | N = 11 3-month and 6 12-month |
|  |  | distal (> 68 um)  U=11 | 0.5734 | N = 10 3-month and 3 12-month |
| S8E | **Two-Way ANOVA (PV intensity)** |  |  | N = 58 3-month and 27 12-month dendrites |
|  | age * bin | F(1, 83) = 17.6625 | < 7e-05 |  |
|  | bin | F(1, 83) = 214.469 | < 2e-24 |  |
|  | age | F(1, 83) = 15.6629 | < 2e-04 |  |
| S8F | **Two-Way ANOVA (PSD95 density)** |  |  |  |
|  | age * bin | F(1, 83) = 5.2503 | 0.0245 |  |
|  | bin | F(1, 83) = 97.7847 | < 2e-15 |  |
|  | age | F(1, 83) = 14.3674 | < 3e-04 |  |
| S8G | **Two-Way ANOVA (PSD95 intensity)** |  |  |  |
|  | age * bin | F(1, 83) = 3.0540 | 0.0842 |  |
|  | bin | F(1, 83) = 30.2794 | < 5e-07 |  |
|  | age | F(1, 83) = 17.3096 | < 8e-05 |  |
| S8H | **Two-Way ANOVA (PSD95 size)** |  |  |  |
|  | age * bin | F(1, 83) = 0.4269 | 0.5153 |  |
|  | bin | F(1, 83) = 6.2350 | 0.0145 |  |
|  | age | F(1, 83) = 1.4862 | 0.2263 |  |
