# Supplementary material for: SynAPSeg: A novel dataset and image analysis framework for deep learning-based synapse detection and quantification
Source: PLoS Comput Biol. 2026 Jul 29;22(7):e1014571. doi: 10.1371/journal.pcbi.1014571 (PMC13432752; doi:10.1371/journal.pcbi.1014571)
Supplement: S3 Table — The StarDist models were used to generate predictions over the benchmark datasets as well as average-sized images from the experimental data presented in Fig 4 (Tilescan) and Fig 5 (Volume). Image size are provided in pixels (Y, X) for 2D data or voxels (Z, Y, X) for 3D volumes. Inference time reflects number of seconds to generate a segmentation, averaged over ten iterations. Note: The reported times encompass the complete end-to-end pipeline utilized by the SynAPSeg framework, including all requisite pre- and post-processing steps. Therefore, these metrics reflect practical, real-world user speeds rather than the isolated inference time of the base StarDist architecture. (DOCX) [file pcbi.1014571.s011.docx]

| **Dataset** | **Image Size** | **Inference Time (s)** |
| --- | --- | --- |
| Benchmark1 | 727, 546 | 0.5635 ± 0.0089 |
| Benchmark2 | 80, 204 | 0.3507 ± 0.0063 |
| Benchmark3 | 8, 128, 128 | 0.5719 ± 0.0117 |
| Tile scan | 16890, 31141 | 285.7344 ± 2.6659 |
| Volume | 23, 2639, 2639 | 90.3889 ± 0.4617 |
