# Supplementary material for: SynAPSeg: A novel dataset and image analysis framework for deep learning-based synapse detection and quantification
Source: PLoS Comput Biol. 2026 Jul 29;22(7):e1014571. doi: 10.1371/journal.pcbi.1014571 (PMC13432752; doi:10.1371/journal.pcbi.1014571)
Supplement: Attachment — Submitted filename: PCOMPBIOL-D-26-00610_Review.docx [file pcbi.1014571.s026.docx]

**SynAPSeg: A novel dataset and image analysis framework for deep** **learning-based synapse detection and quantification**

Summary

The authors develop a deep-learning-based synaptic quantification framework, SynAPSeg, which comprises both an open-source dataset and platform for synaptic segmentation. The authors benchmark their software against other synaptic segmentation softwares such as DeepD3, Cellpose, and StarDist, and integrate those models through a segmentation, validation, and quantification GUI. Notably, the software is compatible with segmentation of shaft synapses, a shortcoming of previous segmentation software. The software appears to perform well relative to human expert annotators. They additionally quantify the PSD95+ puncta of inhibitory hippocampal cells using SynAPSeg, and find that PV intensity correlates with excitatory synapse number. Finally, they describe an age-related reduction in PSD95+ puncta in hippocampal PV neurons. This manuscript develops a useful tool for the field, addressing several gaps in existing technologies. The reviewer recommends this manuscript for publication, with minor revisions.

Major Points

The authors should make it more clear that they are not developing a custom model, just repurposing other models on a new training dataset. This is well-stated in the discussion, but the introduction puts too much emphasis on deep learning architectures for synaptic identification when that is not what’s being developed here.

Given the well characterized role of biological sex in hippocampal spine dynamics (e.g. Woolley et al., 1990), and the inclusion of both sexes in this study, it would be interesting to include a supplement describing sex-dependent differences (or lack thereof) in spine size, density, and intensity across regions and ages.

Minor Points

There is a small typo in line 55. “Though further exacerbate resource demands” should be “though this…” or something similar.

The same line suggests that pooling annotations from multiple experts is a shortcoming of deep-learning techniques, but the authors use this same technique to generate their training dataset. These statements need more qualification.

Figure 1 appears to be missing legend entries for 1D and 1E (in the current version B describes B and C, while C describes D and E).

This manuscript could generally benefit from more statistical testing, for instance in Fig.’s 1C&E, 2B, I, J, &K, and 4D. This would help back up the authors’ claims that there are no significant differences between the StarDist model and expert annotators (as stated in discussion: “performance levels indistinguishable from human experts”). Linear mixed effects models should be used where appropriate to account for nested data.

The authors note that for one dataset, the model’s predictions are 20% larger in size than the expert median. Since the experts provide the only ground-truth for this model this point is non-trivial. Systematic overestimation of object size raises concerns about segmentation accuracy. The authors should elaborate on and statistically quantify this difference.

The authors state that “Importantly, segmentation of these datasets took up to an hour, depending on the annotator and dataset, but just seconds for the trained model.” More specific metrics would be helpful to include in a supplement.

The authors should address the potential caveat that the reduction in PSD95 puncta density may reflect altered PSD95 expression or detectability rather than a true loss of excitatory synapses. It may be helpful to control for PV density via regression as an additional metric confirming that the reduction of PSD95 is a product of aging and not differences in expression across cells.
