# Supplementary material for: SynAPSeg: A novel dataset and image analysis framework for deep learning-based synapse detection and quantification
Source: PLoS Comput Biol. 2026 Jul 29;22(7):e1014571. doi: 10.1371/journal.pcbi.1014571 (PMC13432752; doi:10.1371/journal.pcbi.1014571)
Supplement: Attachment — Submitted filename: ResponseToReviewers.docx [file pcbi.1014571.s028.docx]

**SynAPSeg: A novel dataset and image analysis framework for deep learning-based synapse detection and quantification –Response to reviewers**

We thank the reviewers for their thoughtful comments on our manuscript. We have addressed all comments and believe this has strengthened our manuscript. Please note that in our responses to the reviewer comments below we have underlined parts where next text was added to an existing paragraph.

**Reviewer #1:**

**1) The authors should make it more clear that they are not developing a custom model, just repurposing other models on a new training dataset. This is well-stated in the discussion, but the introduction puts too much emphasis on deep learning architectures for synaptic identification when that is not what’s being developed here. – Reviewer #1 (major point)**

To address this, we have adapted the introduction to better emphasize this distinction *(see line numbers 114-117 and below)*.

“We use this dataset to train models on established deep learning architectures and evaluate the performance of several recent algorithms for synapse segmentation—finding that custom-trained StarDist models show the highest performance on 2D and 3D instance segmentation tasks.”

**2) Given the well characterized role of biological sex in hippocampal spine dynamics (e.g. Woolley et al., 1990), and the inclusion of both sexes in this study, it would be interesting to include a supplement describing sex-dependent differences (or lack thereof) in spine size, density, and intensity across regions and ages. – Reviewer #1 (major point)**

We agree with the reviewer regarding the importance of characterizing sex-dependent differences in hippocampal synapse dynamics. Unfortunately, due to unbalanced sample sizes in this dataset (12-month: 4F, 1M; 3-month: 2F, 4M), a fully powered interaction model (age * sex * region) cannot be run. Specifically, because we only have one 12-month male, a mixed model cannot calculate variance for that group, preventing a statistical comparison of aging trajectories between males and females. However, to address sex differences where possible, we performed the following exploratory comparisons:

1) Within-3-month subset (3-month M vs. 3-month F).

2) Within-Sex Aging Analysis (12-month F vs. 3-month F).

3) Main Effect of Sex (All M vs. All F). We also report the overall main effect of sex across the entire cohort. However, we explicitly note the group imbalance (males skewing young, females skewing old) confounds the sex and age effects.

For each comparison, we ran separate linear mixed models (LMMs) for the 3 dependent variables (puncta size, density, and intensity) with sex and/or age as fixed effects, region as an additive fixed effect, and subject ID as a random effect. Summary statistics have been included in **Table S2** and we make available the full model outputs in the **Supporting Information**. The p-values for the relevant comparisons are summarized below. Across all comparisons the only significant effect was observed in the overall effect of sex on puncta intensity.

|  | Dependent Variables (p-values) | | |
| --- | --- | --- | --- |
| Comparison (effect) | Density | Size | Intensity |
| Within 3-Month Subset: Sex | 0.28 | 0.186 | 0.52 |
| Within Female Subset: Age | 0.505 | 0.845 | 0.905 |
| Overall Cohort Model: Sex | 0.995 | 0.299 | **0.039** |
| Overall Cohort Model: Age | 0.813 | 0.497 | 0.171 |

While the overall effect of sex on PSD95 intensity is interesting, because of the group imbalances this difference could also be influenced by the age group imbalance in the males. In addition, variance component analysis, assessed through the model’s intraclass correlation coefficient (ICC) indicated measurements from the same animals were correlated across regions. For example, we observed an ICC of 0.435 for puncta density, demonstrating that approximately 43.5% of the unexplained variance was attributable to baseline biological differences between individual mice. Collectively, these exploratory results suggest that while global PSD95 features remain stable across age and sex, variability is largely due to region-specific hippocampal architecture and baseline individual-to-individual variation.

We also performed similar analyses to understand the effect of sex for data related to **Figure 5**. We used a linear regression model instead of an LMM since there were no repeated measurements. In addition, we investigated PV dendrite intensity as a dependent variable. However, we only have significant power to evaluate sex-differences within the 3-month group and within females across age groups (3-month: 6M/5F; 12-month: 1M/5F). We have added summary statistics to **Table S2** and report the full results in the **Supporting Information**. Results are summarized below.

1) Within-3-month subset (3-month M vs. 3-month F): no significant differences across density, size, intensity or PV dendrite intensity.

2) Within-Sex Aging Analysis (12-month F vs. 3-month F). Both density and PV dendrite intensity were significantly different (p-values: 0.017 and 0.01 respectively), as indicated by the overall analysis we reported previously.

While there was no significant effect of sex these results confirm that age-dependent changes are preserved within the female cohort.

Results section changes:

*(see line numbers 297-302; text posted below for convenience)*

“Though both sexes were included in the present study, the final cohorts yielded an uneven distribution which failed to reach sufficient statistical power to infer an overall sex-age interaction. We make exploratory statistical comparisons where technically possible: all F vs all M, 3-mo M vs 3-mo F, 3-mo F vs 12-mo F (see **Table S2** for summary statistics and **Supporting Information** for full model outputs). Overall, this analysis did not reveal clearly significant sex-dependent effects.”

*(see line numbers 351-356; text posted below for convenience)*

“Linear regression analysis was performed to isolate the effects of sex and age on PV metrics (**Table S2**). Within the sufficiently powered 3-month cohort (6 males, 5 females), we observed no significant sex differences in density, size, soma intensity, or PV dendrite intensity. Conversely, an age-based comparison restricted to females confirmed our aggregate findings, showing significant changes in both density (p = 0.017) and PV dendrite intensity (p = 0.01) at 12 months compared to 3 months.”

Discussion section changes:

*(see line numbers 494-501; text posted below for convenience)*

“Due to sample size imbalances across groups, a fully powered age-by-sex interaction model could not be conducted. Exploratory comparisons revealed no significant sex differences within the 3-month cohort, nor significant age differences within the female cohort. The most consistent significant effects were seen in a handful of anatomical regions, most notably the MoDG, PoDG, and RadCA3. Collectively, these data suggest that differences are primarily due to region-specific architecture and baseline individual variation. However, it is important to reiterate that due to unbalanced sample sizes we are unable to conclusively address sex-dependent interactions.”

*Methods section changes:*

*(see line numbers 903-915; text posted below for convenience)*

“Linear mixed-effects models (LMMs) were employed to evaluate the effects of age, sex, and anatomical region on PSD95 synaptic puncta metrics (density, size, and intensity) across hippocampal subregions (**Fig 4**). Since multiple anatomical regions were sampled from the same animals, and we observed correlated within-subject baseline measurements (e.g., density’s intraclass correlation coefficient was 0.430), individual animal subjects were designated as a random intercept to account for within-subject biological variability. Due to unbalanced sample sizes within the cohorts, the dataset was insufficiently powered to evaluate a full age x sex interaction. Consequently, the LMMs assessed sex, age, and region as additive fixed effects where possible. To investigate sex-dependent effects related to the analysis of PV dendrites (**Fig 5**), a standard linear regression model was used. In addition to puncta density, size, and intensity, PV dendrite intensity was included as a dependent variable. Similar to the regional analyses, group imbalances precluded a fully powered interaction model. Age and sex were assessed as additive fixed effects.”

**3) This manuscript could generally benefit from more statistical testing, for instance in Fig.’s 1C&E, 2B, I, J, &K, and 4D. This would help back up the authors’ claims that there are no significant differences between the StarDist model and expert annotators (as stated in discussion: “performance levels indistinguishable from human experts”). Linear mixed effects models should be used where appropriate to account for nested data. – Reviewer #1 (minor point)**

We agree with the reviewer and have now performed additional statistical tests to validate these claims and have updated **Table S2** as well as the respective figures and legends to reflect significant differences, as described below.

For Figure 1 C&E, we performed multiple pairwise comparisons between the custom StarDist models and other methods for both the 2D and 3D datasets. This showed that the performance of the StarDist custom models was significantly higher than all other methods across precision, recall, and f1 scores with the only exception being the 2D model’s recall score on the validation fold, which was not significantly different from the custom Cellpose model.

For Figure 2, we ran one-sample t-tests between the annotators and the models for mean IOU (**Fig. 2B**), precision, recall, and F1 scores (**Fig. 2 I-J**) across the three benchmark datasets. In 10 out of these 12 comparisons no significant differences were observed. The exception was comparing the precision and f1 score between the 2D model and the experts in benchmark dataset 1. Though the model did recognize 66 of 69 consensus clusters (recall score: 0.95), it also identified 4 false positives giving it a precision score of 0.89 which was significantly lower than the expert mean of 0.97.

For Figure 4D, we had previously evaluated significance using two-way ANOVA, however we have now updated the results and figure legend to make it more clear that these were run for all dependent variables but there was no significant interaction, so we did not pursue it further.

Results section updates

*(see line numbers 216-221; text posted below for convenience)*

To quantify reliability, we then evaluated human-to-human against human-to-model precision, recall, and F1 scores using consensus labels as ground truth (**Fig 2I-K**). To statistically evaluate the model’s performance relative to human annotators we conducted one-sample t-tests over the IoU, precision, recall, and F1 scores over the three benchmark datasets. No significant differences were observed except when comparing the precision and f1 score between the 2D model and the experts in benchmark dataset 1 (**Table S2**).

*(see line numbers 306-310; text posted below for convenience)*

“Two-way ANOVA of synaptic density indicated 72% of variance was explained by region with no significant effect from age or age x region interaction. The same was observed for intensity, however area did show an overall significant age effect. However, since the interactions were not significant, we did not investigate this further (See **Table S2** for full statistical details).”

Figure 1 legend updates:

“Significant differences between the custom-trained StarDist models and the other models is indicated by a “*” (p<0.05, two-tailed t-test, FDR correction).”

Figure 2 legend updates:

“Precision, accuracy, and recall scores calculated using expert consensus clusters as true positives, and false positives as clusters identified by a single expert or model. For all boxplots, the box extends from the 1st to 3rd quartiles, the center line reflects the median, and whiskers indicate full range. Model IoU, precision, recall, and F1 scores were compared against the expert annotators over the benchmark datasets using one-sample t-tests (**p < 0.01).”

Figure 4 legend updates

“Two-way ANOVAs revealed no significant age x region interaction. Intensity did however show a significant effect of age alone (p=0.0008).”

**4) The authors note that for one dataset, the model’s predictions are 20% larger in size than the expert median. Since the experts provide the only ground-truth for this model this point is non-trivial. Systematic overestimation of object size raises concerns about segmentation accuracy. The authors should elaborate on and statistically quantify this difference. – Reviewer #1 (minor point)**

We agree with the reviewer that this needs to be expanded on. In the initial text we noted this difference was still within the range of expert annotators, indicating the variability between experts. To address this, we performed one-sample t-tests over the benchmark datasets for count, average size, and average intensity of puncta detected by the model, relative to expert annotations. These tests indicated no significant difference in count or size. However, this analysis did reveal a previously unrecognized reduction in mean puncta intensity (p-value = 0.016). The results of these tests are now shown in **Table S2**. We believe this concern is minimized by the fact that feature value disagreements would be applied systematically and therefore should not bias overall group differences.

Results section updates:

*(see line numbers 194-202; text posted below for convenience)*

“Overall, the number, average intensity, and average size of model-predicted labels fell well within the range of human annotations (**Fig S3A-C**). For the 3D benchmark (Dataset 3), we noted that the model’s predictions tended to be about 20% larger in size than the median expert-annotated object, though experts showed high variability and average sizes ranging from 65 to 125 voxels. To follow up on this, we performed one-sample t-tests over the benchmark datasets for count, average size, and average intensity of puncta detected by the model, relative to expert’s annotations. These tests indicated no significant difference in count or size across the three datasets. However, there was a significant reduction in mean puncta intensity for dataset 3 (see **Table S2**).”

Fig S3 legend updates:

*(text posted below for convenience)*

“A-C) Boxplots comparing morphological and intensity features of segmented objects identified by experts and the StarDist model. Features include A) total puncta count, B) mean size (in pixels for datasets 1 and 2; voxels for dataset 3), C) and mean fluorescence intensity. The 2D and 3D StarDist models are indicated by filled and unfilled diamonds, respectively. The count, size, and intensity of objects generated by the model were compared against the expert annotators over the benchmark datasets using one-sample t-tests (*p < 0.05).”

Discussion section updates:

*(see line numbers 451-456; text posted below for convenience)*

“Across all benchmark datasets, the model’s predictions showed strong agreement with the expert consensus, reaching performance levels indistinguishable from human experts. We did, however, observe a subtle, but statistically significant, overall reduction in puncta intensity using our model compared to expert annotators on the 3D dataset (dataset 3). However, we believe this concern is minimized by the fact that feature value disagreements would be applied systematically and therefore should not bias overall group differences.”

**5) The authors state that “Importantly, segmentation of these datasets took up to an hour, depending on the annotator and dataset, but just seconds for the trained model.” More specific metrics would be helpful to include in a supplement. – Reviewer #1 (minor point)**

We agree with the reviewer that providing concrete performance metrics strengthens this claim. To address this, we systematically evaluated the processing time of our custom StarDist models across images of varying sizes and dimensions—ranging from small benchmark crops to large-scale tile scans and 3D volumes. These metrics are now provided in **Table S3**. While we did not conduct formal time-motion tracking for the manual annotations, self-reported times from our expert annotators indicated a minimum of one hour per benchmark dataset. We have updated the manuscript text to refer to this new supplementary data.

| **Dataset** | **Image Size** | **Inference Time (s)** |
| --- | --- | --- |
| Benchmark1 | 727, 546 | 0.5635 ± 0.0089 |
| Benchmark2 | 80, 204 | 0.3507 ± 0.0063 |
| Benchmark3 | 8, 128, 128 | 0.5719 ± 0.0117 |
| Tilescan | 16890, 31141 | 285.7344 ± 2.6659 |
| Volume | 23, 2639, 2639 | 90.3889 ± 0.4617 |

**Supplemental Table 3:** Model inference times**.** The StarDist models were used to generate predictions over the benchmark datasets as well as average-sized images from the experimental data presented in Figure 4 (Tilescan) and Figure 5 (Volume). Image size are provided in pixels (Y, X) for 2D data or voxels (Z, Y, X) for 3D volumes. Inference time reflects number of seconds to generate a segmentation, averaged over ten iterations. Note: The reported times encompass the complete end-to-end pipeline utilized by the SynAPSeg framework, including all requisite pre- and post-processing steps. Therefore, these metrics reflect practical, real-world user speeds rather than the isolated inference time of the base StarDist architecture.

Results section updates

*(see line numbers 221-223; text posted below for convenience)*

"Importantly, while manual annotation of these datasets required upwards of an hour based on annotator self-reporting, the trained model performed complete segmentations in under 2 seconds (**Table S3**)."

**6) The authors should address the potential caveat that the reduction in PSD95 puncta density may reflect altered PSD95 expression or detectability rather than a true loss of excitatory synapses. It may be helpful to control for PV density via regression as an additional metric confirming that the reduction of PSD95 is a product of aging and not differences in expression across cells. – Reviewer #1 (minor point)**

We thank the reviewer for raising the important caveat regarding PSD95 detectability. To address whether the reduction in PSD95 puncta density represents a true loss of synapses or simply altered baseline expression, we incorporated PSD95 intensity, within the dendrite ROI defined by PV expression, as a covariate in a linear regression model to evaluate how PSD95 density is affected by age while controlling for PSD95 intensity. Crucially, the main effect of age on PSD95 density remained significant (p-value: 0.008). This increases our confidence that the age-dependent decrease in PSD95 density is a real biological effect and not just a product of altered PSD95 intensity.

However, we do want to acknowledge that fluorescent imaging will always have the inherent caveat that this reviewer raises. We also want to acknowledge that since we are only looking at PSD95, using this as a proxy for excitatory synapses, there could be populations of synapses that do not express PSD95 which we are unable to quantify. We have adjusted the results to include this analysis and the discussion section to include these caveats of the study.

Results section updates:

*(see line numbers 357-361; text posted below for convenience)*

“To address whether the reduction in PSD95 puncta density represents a true loss of synapses or simply altered baseline expression, we incorporated PSD95 intensity, within the dendrite ROI defined by PV expression, as a covariate in a linear regression model to evaluate how PSD95 density is affected by age while controlling for PSD95 intensity. Crucially, the main effect of age on PSD95 density remained significant (**Table S2**).”

Discussion section updates:

*(see line numbers 523-531; text posted below for convenience)*

“In general, fluorescent imaging and automatic puncta detection offers a promising method to address questions such as synapse density. However, we would like to highlight general limitations of this method, wherein decreased fluorescent signal could lead to detectability issues. Therefore, when possible, orthogonal verification of synapse changes with electron microscopy or electrophysiology will always be advantageous. We acknowledge that we are using PSD95 as a proxy for glutamatergic synapse number. Previous work has suggested that some glutamatergic synapse might lack PSD95 ^47^, consequently it is possible that in the old animals there are greater number of PSD95-negative synapses that we are unable to quantify.”

**Reviewer #2:** 

**7) Fig. S4 and more generally. Were Bassoon puncta detected as reliably as PSD95 given model training mostly focused on the latter. Would be useful to better highlight and quantify utility of the method across labelled proteins. Although they have used different sources of training data, most of the training and (and therefore I assume testing) data is PSD95. Consequently, it is important to know if there is greater error in the non-PSD95 test images, or a uniform accuracy regardless of staining. They state in line 341 that their view is that sample prep is bigger source of variability and so method should be generalizable, but it would be nice to see evidence for this claim. – Reviewer #2 (Major)**

We thank the reviewer for bringing up this insightful distinction. We want to clarify the initial intent with the bassoon puncta figure was for feature demonstration of the quantification pipeline and we do not intend to make any claims about the detection accuracy of different markers because the training dataset has an insufficient sample size to make these claims rigorously. However, this important point is partially addressed by an error we corrected in Figure 2’s legend: it incorrectly stated the marker for benchmark dataset 2 was homer when it is VGluT1 (a presynaptic marker of glutamatergic synapses). The marker identity for this dataset was correctly stated in **Table S1**.

Figure 2 legend update:

“Dataset 2: z-projection showing *in vitro* preparation where VGluT1 puncta were labeled by immunohistochemistry.”

**8) For Figure 5, where on the dendrite were images taken? There is evidence that synapse density onto PV cells drops as you move further from the soma (e.g Morabito et al., Neuron 2025). How did they account for this effect and (ideally) can they replicate it? They state that size and length of dendrites not different, but where on the dendrite might also be important. – Reviewer #2 (Major)**

We thank the reviewer for raising this excellent point. While the distance-dependent drop in synapse density has been elegantly described in cortical PV cells (Morabito et al., 2025), to our knowledge, a similar gradient remains uncharacterized in CA1 PVs. Prompted by this comment, we performed a follow-up analysis to determine if a similar spatial gradient exists within our dataset (see new **Supplemental Figure 7** below).

To do this, we restricted our analysis to dendrites with a visibly attached soma and quantified the distance of each analyzed segment from the cell body. This subset represents 76% of the original data and 32% of this subset were dendrites from 12-month animals. Because the size of the imaging volumes was around 11x244x244 µm^3^ and were centered on the pyramidal layer, this dataset is biased towards proximal/intermediate dendrites rather than far-distal segments extending into the SLM. More precisely, 96% of the total dendritic length analyzed fell within 100 µm of the soma (the boundary defined as 'distal' by Morabito et al.). However, to analyze whether distance from soma affected synapse density we set a “distal” threshold as the 75^th^ percentile of the distribution of distances from soma (68 µm). The proximal threshold was set to the same value proposed by Morabito et al (40 µm). We acknowledge this definition is arbitrary and more anatomically defined definition may extend past the SLM for categorization as distal, which is not represented in our dataset.

Within this domain, we observed no correlation between synapse density and distance from the soma for the 3-month animals and a weak but significant positive correlation in the 12-month group (**Fig S7C**). We also analyzed density in these distance bins using the animal averages and we also observed a significant reduction in PSD95 density for the 12-month group in the proximal and intermediate dendrites (**Fig S7E**). While these analyses largely suggest that dendritic location does not influence the density metrics reported in this study, investigating whether a proximal-distal gradient shapes PSD95 density in hippocampal SLM-projecting dendrites remains an exciting avenue for future study.

We have updated the manuscript text to more explicitly define the dendritic population analyzed, included **Supplemental Figure 7** to better illustrate our methodology, and added the statistical comparisons to **Table S2**.

*The following sections were added to the manuscript:*

**Supplemental Figure 7.** PSD95 puncta density along proximal-distal axis of PV CA1 dendrites. **A)** Representative maximum intensity z-projection of whole volumes showing PV (magenta) dendrites and PSD95 (green) puncta in 3-month (left) and 12-month (right) animals. Scale bar represents 20 µm. **B)** The origin and end of dendrites were similar in distance from soma between 3-month (red) and 12-month (gray) age groups. **C)** Correlation between distance from soma and PSD95 puncta density. Data points represent discrete 2 µm dendritic segments. No correlation was observed for 3-month animals while a weak but significant positive correlation was observed in 12-month group (Pearson; r=0.0, p=0.92 3-month; r=0.25, p < 0.001 12-month). The correlations were significantly different (Fisher r-to-z; z=4.44, p < 0.001). **D)** Histogram showing the distribution of distance from soma for the dendrites analyzed. **E)** Bar plots show puncta density in proximal, intermediate, and distal dendritic segments. Significant differences in PSD95 density (Mann-Whiteny U test) were observed between age groups in the proximal (*p=0.0103) and intermediate (**p=0.0071) segments. Data points represent per-animal means.

Results section update:

*(see line numbers 373-390; text posted below for convenience)*

“Because synapse density onto PV interneurons has been shown to vary along the proximal-distal axis in other brain regions ^63^, we analyzed whether a spatial gradient existed in our dataset. This was performed on a subset (76% of the original dendrites) where the PV-positive dendrite was visibly attached to a soma. Since our images were centered on the CA1 pyramidal layer **(Fig S7A)**, this dataset comprises dendrites within the SO and SR subregions. On average, the origin and end of each dendrite was located 33.76 and 62.81 µm from the soma, respectively, and were similar between age groups **(Fig S7B)**. To analyze density with respect to distance from soma, dendrites were broken up into discrete 2 µm dendritic segments. We observed no correlation between distance from soma and PSD95 puncta density for 3-month animals while a weak but significant positive correlation was observed in the 12-month group **(Fig S7C)**. The correlations were significantly different. Since 96% of the analyzed dendritic length fell within 100 µm **(Fig S7D)**, we interpret this dataset to be primarily comprised of proximal/intermediate dendritic segments. However, we analyzed PSD95 density within subsets of the data where distal segments were defined as over 68 µm from the soma (25% of the data), proximal as under 40 µm (36% of the data), and intermediate segments in between (39% of the data). Consistent with what we show for the whole dataset **(Fig 5)** we observed a significant reduction in PSD95 density in the 12-month group in the proximal and intermediate dendrites **(Fig S7E)**.”

Methods section update:

*(see line numbers 881-890; and shared methods described for response to comment #9 below)*

**9) Additional analysis – not essential but very useful and enhances impact. Figure 5 and S6. Related to the point above and the analysis of PSD95 clusters. It seems that there is a reasonable amount of PV variability within a dendrite as well as between dendrites/animals. If they were to trace along the dendrite and plot PSD95 density and intensity as a function of PV intensity, do they co-vary? Given PV is a calcium buffer you could imagine it might be biased towards regions of high synapse density or stronger synapses and therefore calcium. This would be an interesting finding that could enhance the impact of the study. – Reviewer #2 (Major)**

We are grateful to the reviewer for this fascinating question which we have now followed up on. While somatic PV expression has been correlated with increased excitatory synapse density (Donato et al. 2013), we are not aware of any articles investing whether this relationship holds up within discrete dendritic compartments. We therefore performed the analysis shown below in new **Supplemental Figure 8** (see below).

To investigate this question, we analyzed the subset of dendrites discussed above (where cell body was visibly attached) and extracted PV intensity, PSD95 density, PSD95 intensity, and PSD95 size along the dendritic segment within 2 µm bins. We then sampled and averaged these features in the highest and lowest PV intensity bins within each dendrite. Repeated measure ANOVAs indicated an overall effect of PV bin (high vs low PV intensity) and age on PSD95 density and intensity, with higher PV regions having brighter and more dense PSD95 puncta. There was no significant effect of age on PSD95 size. In addition, there was a significant age x PV bin interaction for PV intensity and PSD95 density, but not PSD95 intensity or size.

Overall, these results indicate that PSD95 density and intensity are significantly higher in regions with high PV intensity, while also replicating the age-dependent differences in PSD95 density and PV intensity shown in **Figure 5**. We have included this interesting observation into our discursion section and plan to follow up on these findings in the future.

**Supplemental Figure 8.** PSD95 puncta density and intensity co-vary with PV intensity. **A)** Confocal maximum intensity z-projection of a PV positive dendrite from 3-month animal. White circles indicate the positions of PSD95 puncta. Scale bar indicates 5µm. **B)** Same as A but from a 12-month animal. **C)** Line plots showing PV intensity (magenta), PSD95 puncta density (black), and PSD95 puncta intensity (green) extracted within a 2µm sliding window along the length of the dendrite in A. Individual traces were smoothed using a gaussian kernel (sigma=3.0). **D)** Same as C but for the dendrite in B. **E-H)** Quantitative analysis of synaptic parameters stratified by PV expression level (low vs. high bins). Data were binned within discrete 2µm segments, sampled such that one high and one low PV bin were taken for every 10µm of dendrite, and averaged within dendrites. Mean binned values are compared for **E)** PV intensity, **F)** PSD95 density, **G)** PSD95 intensity, and **H)** PSD95 size across 3-month (red) and 12-month (grey) dendrites. Statistical comparisons were made between high and low bins within each age group as well as between group comparisons within high or low bins using repeated measure ANOVAs. Line plots represent individual dendrites.

Results section update:

*(see line numbers 391-403; text posted below for convenience)*

“To investigate the spatial relationship between PV expression and excitatory synapses along individual dendrites, we examined localized PV intensity and PSD95 puncta characteristics in 3-month and 12-month animals **(Fig S8A, B)**. Correlation analysis using a sliding window to analyze properties at points along the length of individual dendrites demonstrated a potential relationship between PV intensity and PSD95 puncta properties **(Fig S8C, D)**. To quantify this relationship, dendrites were divided into discrete 2 µm dendritic segments and stratified into 'high' and 'low' PV expression bins **(Fig S8E)**. We found that high PV intensity bins also had increased PSD95 density and PSD95 intensity relative to low bins **(Fig S8F, G)**. Furthermore, repeated measures ANOVA revealed significant main effects of age, with 3-month animals generally exhibiting higher overall PV intensity, PSD95 density, and PSD95 intensity compared to 12-month animals (**Table S2**). Notably, while PSD95 density and intensity co-varied with PV levels and showed a significant age effect, PSD95 puncta size showed a small but significant effect of bin but not age **(Fig S8H)**.”

Discussion section update:

*(see line numbers 517-522; text posted below for convenience)*

“Curiously, we observed hotspots of high and low PV expression along the dendrite which, in an age-independent manner, showed varied PSD95 puncta density and intensity. Our analysis showed there are dendritic hotpots of glutamatergic inputs, in line with our nearest neighbor analysis which indicated synapses close in space are more similar to one another. The functional implication of this spatial relationship and how it relates to PV expression levels remains unexplored.”

Methods section update:

*(see line numbers 881-902; text posted below for convenience)*

“To investigate spatial relationships between the properties of PSD95 puncta along dendrites, we analyzed a subset of the data where the dendrites were visibly connected to PV-positive soma. Overall, this subset represented 76% of the original data set. Dendritic length was extracted using skeletonization as described above and PSD95 puncta locations were mapped along the dendritic shaft. For analysis of PSD95 density in proximal, intermediate, and distal segments, we used a distance threshold based on the distribution of distances from soma after dividing each dendrite up into 2 µm segments. We set the 25^th^ and 75^th^ percentiles of this distribution as thresholds to classify segments as being proximal or distal, 40 and 68 µm respectively. We then analyzed each dendrite independently, calculating the density in these proximal, intermediate, or distal segments.

For analysis of PV covariation with PSD95 properties, the same subset of data described above was used. To generate continuous spatial profiles of PV intensity, PSD95 puncta density, and PSD95 puncta intensity, these properties were extracted using a 2 µm sliding window along the length of the dendrite. The resulting individual traces were smoothed using a Gaussian kernel (sigma = 3.0). For quantitative categorical analysis, data were binned into discrete 2 µm segments based on PV expression levels, categorized as either 'low' or 'high' PV intensity. To ensure uniform sampling across varying dendrite lengths, one high PV bin and one low PV bin were sampled for every 10 µm of dendrite. These binned values were then averaged within each individual dendrite. Statistical comparisons of synaptic parameters (PV intensity, PSD95 density, PSD95 intensity, and PSD95 size) were conducted using repeated measures ANOVAs to assess differences between high and low bins, between age groups (3-month vs. 12-month), and the interaction between bin and age.”

**10) To improve the ease of interpretation, it would be helpful if they could explain what the computer vision metrics (IoU etc) means in the context of their experiments and how to interpret the graphs in F1 for non-experts. – Reviewer #2 (Minor)**

We agree that translating these computer vision metrics into practical biological terms is essential for ensuring the framework is accessible to a broader neuroscience audience. We have expanded the Results section corresponding to Figure 1 to explicitly define the IoU, precision, recall, and F1 scores – and how these inform the interpretation of the graphs.

Results section update:

*(see line numbers 167-173; text posted below for convenience)*

“Performance was evaluated using Intersection over Union (IoU) scores which measure the spatial overlap of the model’s predicted labels and the ground truth labels. We used a standard IoU threshold of 0.5 to classify detections as true positives, false positives, and false negatives and calculate recall, precision, and F1 scores. High recall indicates the model misses very few actual synapses. High precision indicates the model rarely mistakes background noise or artifacts for synapses (low false positives). F1 is the harmonic mean of precision and recall, providing a measure of overall performance.”

**11) Figure S4 and associated text. How were dendrites determined? While I don’t doubt most synapses were aspiny, unless you can see parent dendrites then it is hard to assess this. – Reviewer #2 (Minor)**

We thank the reviewer for raising this important point. The dendritic regions were manually delineated using the GAD67 signal, which clearly labeled the morphology of the parent dendrites. Because our primary goal for this figure was to demonstrate how the platform performs analysis within user-defined semantic regions, we originally omitted the GAD67 channel from the composite image in **Fig S4** (shown below with updated legend) to avoid visual clutter and highlight the synaptic puncta. We agree that clarifying this methodology is important, and we have revised the figure and legend to show the GAD67 channel and explicitly state that the GAD67 signal was used to determine the dendritic ROIs.

**Supplemental Figure 4**. Implementation of the SynAPSeg framework for 3D colocalization analysis of pre- and postsynaptic markers. A) Maximum intensity z-projection of a Gad67 (cyan)-expressing putative inhibitory neuron in neuronal culture where bassoon (green) and PSD95 (magenta) were labeled by immunocytochemistry (left). The Gad67 channel was used to define dendritic ROIs (right). Blue and red outlines indicate manually defined primary and secondary dendritic segments, respectively. B) High-magnification views of segmented bassoon (left) and PSD95 (right) puncta. C) Visualization of colocalized puncta representing putative synapses identified via 3D object-based colocalization in SynAPSeg. D) Barplots (left) show the percentage of PSD95 puncta colocalized with bassoon (relative to the total number of PSD95 puncta) and the density of synapses (right) for primary and secondary dendrites. E) Demonstration of capability to extract features from individual synaptic objects, illustrated by the distribution of colocalized PSD95 puncta size (total number of voxels) across dendritic ROIs.

**12) Line 55 suggests that pooling annotations from multiple experts is a shortcoming of deep-learning techniques, but the authors use this same technique to generate their training dataset. These statements need more qualification. – Reviewer #2 (Minor)**

We thank the reviewer for pointing out this apparent contradiction. Our intention was not to criticize consensus pooling as a methodological flaw, but rather to highlight it as a significant resource bottleneck. Pooling annotations is a rigorous strategy to mitigate human subjectivity and establish a reliable ground truth—which is why we employed it for our benchmark dataset. However, because it multiplies the manual labor required per image, it exacerbates the 'time cost' problem of dataset generation. We have revised the text to clarify this distinction"

Introduction section update:

*(see line numbers 73-75; text posted below for convenience)*

“Consequently, annotations from multiple experts are frequently pooled to generate a consensus. Though this represents a highly rigorous method for establishing ground truth when there is uncertainty among experts, it further exacerbates resource demands.”

**13) Line 225. Wording. I would suggest density of protein, or intensity of staining but not intensity of protein. Also, they mention intensity in text but plot size. Could be helpful to explain how the two are related in small structures such as synapses. Also, in the legend need to explain how size is calculated.**

We thank the reviewer for catching these important points. We have amended the figure legend and results sections to clarify these issues.

Results section update:

*(see line numbers 258-262; text posted below for convenience)*

“Beyond accurate density measurements, a key advantage of instance segmentation is the ability to extract features from individual puncta. This is particularly valuable given that the abundance of postsynaptic proteins, such as PSD95 and GluA1 measured by the intensity of their fluorescent signal, have been shown to positively correlate with synaptic strength^1,42^.”

Supplemental Figure 4 legend update:

“E) Demonstration of capability to extract features from individual synaptic objects, illustrated by the distribution of colocalized PSD95 puncta size (total number of voxels) across dendritic ROIs.”

**14) In methods they mention 4 human experts, yet figures show 5 annotators.**

We thank the reviewer for pointing out this point of confusion. For the training dataset, annotations were performed by four annotators. For the benchmark datasets five annotators labeled the benchmark datasets. We have amended the text to make the distinction between annotations done for the training and benchmark datasets clearer.

Methods section update:

*(see line numbers 554-556; text posted below for convenience)*

“To generate the labels for the training dataset, pixel-wise manual annotation was conducted by four human experts.”

Results section update:

*(see line numbers 192-193; text posted below for convenience)*

“For each image in the benchmark datasets, five human experts provided independent, pixel-wise instance segmentation annotations.”
